# Automated multigroup outlier identification in molecular high-throughput data using bagplots and gemplots

**DOI:** 10.1186/s12859-017-1645-5

**Published:** 2017-05-02

**Authors:** Jochen Kruppa, Klaus Jung

**Affiliations:** Institute for Animal Breeding and Genetics, University of Veterinary Medicine Hannover, Foundation, Bünteweg 17p, Hannover, D-30559 Germany

**Keywords:** Bagplot, Gemplot, High-dimensional data, Outlier, Principal component analysis

## Abstract

**Background:**

Analyses of molecular high-throughput data often lack in robustness, i.e. results are very sensitive to the addition or removal of a single observation. Therefore, the identification of extreme observations is an important step of quality control before doing further data analysis. Standard outlier detection methods for univariate data are however not applicable, since the considered data are high-dimensional, i.e. multiple hundreds or thousands of features are observed in small samples. Usually, outliers in high-dimensional data are solely detected by visual inspection of a graphical representation of the data by the analyst. Typical graphical representation for high-dimensional data are hierarchical cluster tree or principal component plots. Pure visual approaches depend, however, on the individual judgement of the analyst and are hard to automate. Existing methods for automated outlier detection are only dedicated to data of a single experimental groups.

**Results:**

In this work we propose to use bagplots, the 2-dimensional extension of the boxplot, to automatically identify outliers in the subspace of the first two principal components of the data. Furthermore, we present for the first time the gemplot, the 3-dimensional extension of boxplot and bagplot, which can be used in the subspace of the first three principal components. Bagplot and gemplot surround the regular observations with convex hulls and observations outside these hulls are regarded as outliers. The convex hulls are determined separately for the observations of each experimental group while the observations of all groups can be displayed in the same subspace of principal components. We demonstrate the usefulness of this approach on multiple sets of artificial data as well as one set of gene expression data from a next-generation sequencing experiment, and compare the new method to other common approaches. Furthermore, we provide an implementation of the gemplot in the package ‘gemPlot’ for the R programming environment.

**Conclusions:**

Bagplots and gemplots in subspaces of principal components are useful for automated and objective outlier identification in high-dimensional data from molecular high-throughput experiments. A clear advantage over other methods is that multiple experimental groups can be displayed in the same figure although outlier detection is performed for each individual group.

**Electronic supplementary material:**

The online version of this article (doi:10.1186/s12859-017-1645-5) contains supplementary material, which is available to authorized users.

## Background

Modern molecular biology produces high-dimensional data en masse where the number of features is much larger than the sample size of the study or the experiment. Some typical examples are (metric) gene and protein expression data observed with DNA microarrays [1, 2], next-generation sequencing (NGS) [3] or proteomics techniques such as mass spectrometry [4, 5] or 2-D gel electrophoresis [6]. In the case of microarray and proteomics experiments, expression data are generally continuous fluorescence or intensity values [7, 8], while NGS produces expression data as read counts [9] or also as continuous quantities [10]. Other examples of high-dimensional data in molecular biology are methylation levels that can also be observed with microarrays [11] and NGS [12], or data from binding experiments such as chromatin immunoprecipitation (ChIP) [13] in combination with microarrays or NGS, or affinity purification mass spectrometry (AP/MS) [14].

Typical questions analyzed in high-dimensional data focus on the correlation of expression levels with experimental factors or with patient data. For example gene expression data is usually analyzed to detect differentially expressed genes between two levels of an experimental factor (e.g. treatment versus control) or between two patient groups [15–17]. Moreover, high-dimensional expression data is often used to train classifier and regression models to predict therapy outcome [18] or survival [19, 20].

Throughout this work, the term ‘observation’ is used to specify the measured data of one experimental unit (e.g. one patient), and the term ‘sample’ means a set of experimental units. Statistical and bioinformatics analyses of the above questions are usually very sensitive to a single observation, i.e. its addition or removal can seriously affect the results. For example when searching for differentially expressed genes or training a classifier model, individual observations can have a strong impact on the ranking of genes or the estimates of the classifier’s performance. Therefore, quality control of the raw data often involves their inspection with respect to extreme observations. An extreme observation in an experimental group could either be a) regular but just extreme observation, b) a mislabeled observation or c) the consequence of an incorrect measurement. In the first case robust methods can help to decrease or downweigh the outlier’s impact [21–23]. If the last case becomes obvious, the observation can be removed from data analysis. In the case of a mislabeled observation this can be corrected. In either case, the identification of outliers can help to continue with a correct analysis.

The most widely used tools for outlier detection in molecular high-throughput data are hierarchical clustering [24] and principal component analysis [25]. In hierarchical clustering all observations are plotted in a tree, where similar observations appear at branches near to each other and unsimilar observations appear at branches farther away from each other. Principal component analysis (PCA) performs first a dimension reduction so that the high-dimensional data can be represented in a two- or three-dimensional plot while a certain proportion of variance of the original data is maintained. In a PCA plot similar observations group together and unsimilar observations appear again farther away from each other. A useful tool for interactive 3D-visualization of principal components is given by the R-package ‘GGobi’ [26]. Using hierarchical cluster plots and PCA plots, outliers can be identified by visual inspection which is, however, a subjective decision of the researcher. When using hierarchical clustering, the ‘single linkage’ approach usually performs best to identify outliers [27].

Besides visual methods, some automated approaches have been proposed, several of them were reviewed by Egan and Morgan [28] and Zimek et al. [29]. Methods for automated outlier detection based on robust PCA were for example proposed by Model et al. [30], Hubert et al. [31] as well as Filzmoser and Todorov [32]. These methods focus, however, only on the data of one experimental group. Because in these approaches, outlier detection and graphical representation are linked, multiple experimental groups can not be displayed in the same plot.

In order to detect outliers individually in multiple experimental groups but to display the results in the same plot we propose an approach that is also based on PCA. In particular, PCA is first performed for the whole data set and bagplots are then used individually for each experimental group to identify outliers in the space of the first two principal components. In addition, we newly present the ‘gemplot’ - the three-dimensional version of the bagplot - and propose to use this plot for automatically identification of outliers in the space of the first three principal components.

In the methods section, the basic principal of PCA as well as the concepts of bagplot and gemplot are detailed, and the example data is described. In the results section we demonstrate the usefulness of our approach on multiple data sets of artificially constructed principal components and one set of public gene expression data from a kidney cancer study. We close this work with some conclusions.

## Methods

In this section, we first describe the basic idea of PCA and detail next the idea of outlier detection by means of boxplots, bagplots and the new gemplot. Finally, we present the example data that we use for illustrating our approach.

### Principal component analysis

Genomic data typically follows a high-dimensional setting where the number of molecular features *d* is much larger than the sample size *n*. In order to visualize such data, dimension reduction is usually used, for example by means of PCA which has been widely used in genomics data analysis. PCA projects the data into the space of orthogonal principal components. Each component represents a certain proportion of variance of the original data. Thus, in many cases the first two components represent more than 50% of the variance of the original data and are sufficient to display the location of the single observations to each other and to detect extreme observations. Raychaudhuri et al. [33] show microarray data, where the first two components present even more than 90% of the original variance, and Shieh and Hung [34] also point out that a small number of components is usually needed to explain most of the variance in high-dimensional settings. Cangelosi and Goriely [35] show several data sets of gene expression data, where the first three components represent 80% of the original variance. Sharov et al. [36] show gene expression data, where outliers become obvious when plotting the first three components. More precisely, the original (*n*×*d*) data matrix *X* is transformed into *d* orthogonal components given by the columns of *Z*=*X*·*A* [37]. It can be shown that the columns of the (*d*×*d*) transformation matrix *A* are the eigenvectors corresponding to the eigenvalues *λ*_*j*_ (*j*=1,…,*d*) of *S*, where *S* is the sample covariance matrix of *X*. Moreover, the eigenvalue *λ*_*j*_ is equal to the variance of the *j*th principal component. Hence, $V_{j}=\lambda _{j} / \sum \lambda _{j}$ represents the proportion of variance that is explained by the *j*th component. For the two- and three-dimensional representation of the original data one can than use the first two or three components, respectively, given by the first three columns of *Z*, i.e. *z*_1_, *z*_2_ and *z*_3_.

### Boxplot, bagplot and gemplot

The box-and-whiskers-plot, mostly just called boxplot, is one of the most frequently used graphical tools to display the quantiles of a metric feature. It consists of a box whose lower and upper limits represent the 25- and 75%-quantile of the observed data and a bar within the box represents the median, i.e. the 50%-quantile. Thus the box includes 50% of the data points. In its standard version, the whiskers go from the 75%-quantile to the maximum of the data and from the 25%-quantile to the minimum of the data, respectively. In order to detect outliers the whiskers are often limited by the so called fence, where their maximal length is only allowed to be 1.5 times the interquartile range (the difference between 25- and 75%-quantile). Each observation that is beyond the fence is drawn as a single dot and is regarded as an outlier.

The two-dimensional extension of the boxplot, the bagplot, was first presented in 1999 by Rousseeuw et al. [38]. Instead of a box, a convex hull - called the bag - is contructed so that 50% of the data points in the center of the two-dimensional point cloud are included in this bag. The bag is determined by making use of the concepts of halfspace location depths and depth regions. For each point *θ* in the two-dimensional space, $\phantom {\dot {i}\!}ldepth(\theta,(z_{1}^{i}, z_{2}^{i}))$ is the smallest number of data points $(z_{1}^{i}, z_{2}^{i})\phantom {\dot {i}\!}$, *i*=1,…,*n*, contained in any closed halfplane with margins through *θ*. In our case, the coordinates of the data points $(z_{1}^{i}, z_{2}^{i})\phantom {\dot {i}\!}$ are given by the first two principal components. The depth region *D*_*k*_ is then given by the set of all *θ* with *l* *d* *e* *p* *t* *h*≥*k*. If we denote the number of data points in *D*_*k*_ by *#* *D*_*k*_, we need to find that *k* ^∗^ for which $\#D_{k^{*}}\leq \lfloor n/2 \rfloor < \#D_{k^{*}-1}\phantom {\dot {i}\!}$. The bag can than be found by interpolating between the sets $D_{k^{*}}\phantom {\dot {i}\!}$ by $\phantom {\dot {i}\!}D_{k^{*}-1}$. As center of the data, the depth median *T* is given by that *θ* with the highest *ldepth*. If there are multiple such *θ*, *T* is given by their center of gravity. Similar to the role of the whiskers in a boxplot a loop is constructed around the bag, where observations outside this loop are regarded as outliers. In order to construct the loop, one first generated the fence by inflating the bag relative to *T* by a factor of 3. The loop contains all data points within the fence that don’t belong to the bag. For the detailed description of the construction of a bagplot we refer to the original work of Rousseeuw et al. [38]. An algorithm for the fast computation of halfspace location depths is for example given in Miller et al. [39].

Rousseeuw et al. already pointed out that the bagplot is, in principle, defined in any dimension. Instead of halfplanes, halfspaces are then used to determine the halfspace location depths. Since it is computationally extreme and also hard to visualize bagplots in higher dimensions, there was not yet an implementation for the three-dimensional case. Here, we present this three-dimensional version for the first time and call it ‘gemplot’ due to the similarity of its appearance as gemstones. We also provide the software package ‘gemPlot’ for the R programming environment available at https://github.com/jkruppa/gemPlot. In order to determine the sets *D*_*k*_ in the three-dimensional space, our implementation uses a three-dimensional array that lays a three-dimensional grid across the data points. For each grid point *θ*, $\phantom {\dot {i}\!}ldepth(\theta,(z_{1}^{i}, z_{2}^{i}, z_{3}^{i}))$ is calculated. Thus, it is also possible – though time-consuming – to calculate the gemplots in more than three dimensions. A special graphical trick to make the visualization of gemplots possible is the use of transparent colors. Thus, the depth median and the bag (‘inner gem’) are still visible when drawing the loop (‘outer gem’). When the median is not a single point, we found that it is more appropriate not to display the center of gravity but also a further gem which is made up by another convex hull. In our implementation, the gemplot is drawn in an interactive device so that the researcher can rotate the gemplot with the computer mouse and can also zoom in and out. Furthermore, our implementation can also determine outliers in subspaces of more than three dimensions (without visualization), which is however computational very expensive.

## Data examples

We demonstrate the practicability of bagplots and gemplots for outlier detection in high-dimensional data on two sets of artificially generated principal components, different data sets of artificial gene expression data, as well as on a data set originating from an RNA-seq experiment in a tumor study.

### Artificial data examples

In order to make users familiar with our approach and it’s behavior we generated two simple data sets which directly represent the values of three principal components. In both artificial data sets, data for two study groups were generated. In the first example the first three principal components, *z*_1_, *z*_2_ and *z*_3_, were drawn from the same normal distribution within each group. Here, the order to the three components was defined arbitrarily. For study group one the principal components were drawn from $\mathcal {N}(\mu =-3,\sigma =1)$ and from $\mathcal {N}(\mu =3, \sigma =1)$ for the second study group. The samples size for each group was *n*=100. Since outliers are more frequent in skewed distributions, some principal components in the second example data set were drawn from the exponential distribution. In particular, *z*_2_ in one study group and *z*_1_ and *z*_3_ in the other study group were drawn from *E* *x* *p*(*λ*=4).

In order to introduce outliers into both examples, we changed the coordinates of arbitrarily chosen observations. In the example with only normally distributed components, we changed the coordinates of observation 3 in the first study group to (−6,−6,−6) and of observation 8 in the second group to (6,6,6). In the example with non-normal components, we set the coordinates of observation 73 and 87 in the first group to (3,4,25) and (3,10,4), respectively. Thus, the outlying character of observation 73 manifests itself in the third component, and that of observation 87 in the second component.

In a further example of artificial data, we wanted to study how outliers are detected by our approach when principal components are derived from high-dimensional expression data. Therefore, we simulated in two different settings expression data from the multivariate normal distribution, $\mathcal {N}_{d}(\mu,\Sigma)$, for *d*=1000 genes and *n*=100 samples. In both settings we used an (*d*×*d*) autoregressive covariance matrix *Σ* with entries *σ*_*ij*_=*ρ* ^*τ*·|*i*−*j*|^ each representing the covariance between gene *i* and *j* (*i*,*j*=1,…,*d*). For *i*=*j*, we chose *σ*_*ij*_=1 for all genes. Setting 1 included data for one experimental group, and with three outliers. The construction of the mean vector *μ* for the non-outlying observations, and the mean vectors *μ*_1_, *μ*_2_, *μ*_3_ for the outliers is specified in Table 1. The mean vectors for the outliers were constructed so that each outlier represents a different group and appears at one direction of the first three principal components. In setting 2, a second group is included also with three outlying observations. Non-outlying observations and outliers for the second group are constructed by mean vectors *κ*, *κ*_1_, *κ*_2_, *κ*_3_, also specified in Table 1. In each setting, we varied the parameter *τ* for the generation of the covariance matrix, as well as the parameter *fc* (for fold change) that reflects how far away an outlier is from its group (Table 1).

**Table 1:** Contruction of simulation parameters for artificial gene expression data from the multivariate normal distribution

Setting	Group 1	Group 2	Outliers	Parameter values
1	*μ*=0_*d*_	-	*μ*_1_=*f* *c*⊗*J*_*d*_	*f* *c*=0.5,0.75
			*μ*_2_=(*f* *c*,−*f* *c*)^*T*^⊗*J*_*d*/2_	*ρ*=0.75
			*μ*_3_=(*f* *c*,−*f* *c*,*f* *c*,−*f* *c*)^*T*^⊗*J*_*d*/4_	*τ*=0.01,0.05
2	*μ*=0_*d*_	*κ*=5·*J*_*d*_	*μ*_1_=*f* *c*⊗*J*_*d*_	*f* *c*=1.0,1.5
			*μ*_2_=(*f* *c*,−*f* *c*)^*T*^⊗*J*_*d*/2_	*ρ*=0.75
			*μ*_3_=(*f* *c*,−*f* *c*,*f* *c*,−*f* *c*)^*T*^⊗*J*_*d*/4_	*τ*=0.05,0.1.,0.2
			*κ*_1_=*κ*+(*f* *c*⊗*J*_*d*_)	
			*κ*_2_=*κ*+((*f* *c*,−*f* *c*)^*T*^⊗*J*_*d*/2_)	
			*κ*_3_=*κ*+((*f* *c*,−*f* *c*,*f* *c*,−*f* *c*)^*T*^⊗*J*_*d*/4_)	

Setting 1 represents data for one group with three outliers. Setting 2 represents data for two group, each with three outliers. The last columns shows the simulation parameters that are varied. The mean vectors in group 1 and group 2 for regular observations are given by *μ* and *κ*, and those mean vectors for outlying observations are given by *μ*_1_, *μ*_2_, *μ*_3_ and *κ*_1_, *κ*_2_, *κ*_3_. In this notation, *J*_*L*_ denotes a vector of ones of length *L* and ⊗ denotes the symbol for the Kronecker product

### RNA-seq data of kidney tumors and controls

In the above simulated principal components, no within group correlation was given. In real data with multiple experimental groups, it may happen that principal components within a study group are correlated, although the principal components for the whole data are uncorrelated. In the simulated data principal components were generated uncorrelated whithin each group. We therefore considered also one example of real data from an RNA-seq experiment. As will be demonstrated in the analysis of these data, correlation between the components whithin a group can have a strong impact on the outlier detection. The related experiment was performed as part of The Cancer Genome Atlas Project [40] and involved samples from kidney renal clear cell carcinomas. The data set contains expression data of *d*=20531 genes in *n*=144 tissue samples from either non-tumor (control) tissue or tumor tissue. The total sample divided into 72 observations from each tissue type. The data is available in the R-package ‘SimSeq’. The experiment was originally conducted to find pathways and genes which take part in development of kidney cancer, or renal cell carcinomas (RCC), which are a common group of hemotherapy-resistant cancer types and therefore of special interest for the search of gene mutations [40].

## Results

In this section we show the results of outlier detection in the example data sets and the simulation study. In detail, we show the different results that bagplots and gemplots produce and add a comparison with outlier detection by means of hierarchical clustering.

### Analysis artificial principal components

**Fig. 1:**
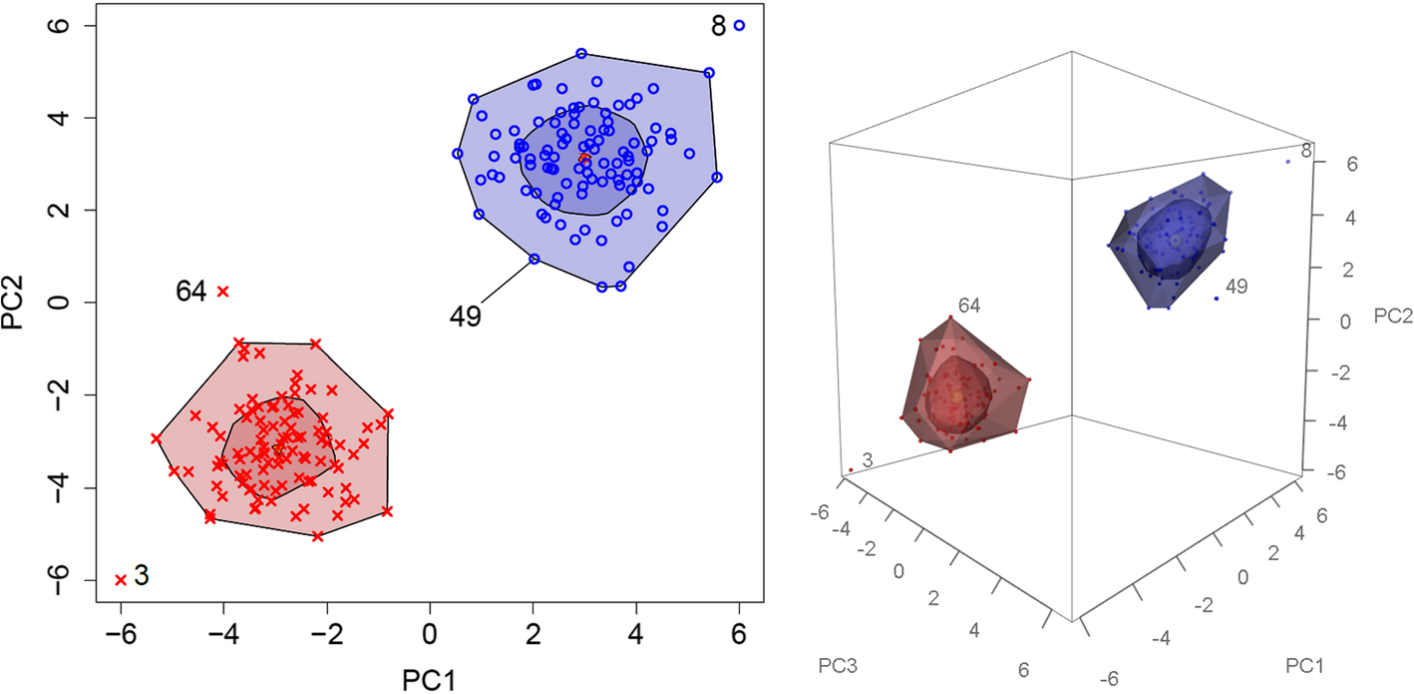
Bagplots and gemplots under normaly distributed prinicipal components. Bagplots (*left*) and gemplots (*right*) for identifying outliers in the space of the first two or three principal components, respectively. The plots consist of inner bags (or *inner* gemstones) that contain 50% of the samples and an outer loop (or *outer* gemstone). Sample outside the loop or gemstones are flagged as outliers. While multiple experimental groups can be displayed in the same subspace of principal components, outlier detection can be performed separately for each group. Switching from two to three dimensions, new outliers can be found (sample 49) while other sample disappear as outliers (sample 64)

In the first example with normally distributed principal components, the outlying observations 3 and 8 were detected in the two-dimensional analysis using a bagplot for each of the two study groups (Fig. 1). In addition, another outlier, observation 64, was detected in the first group. When the analysis if restocked by the third principal component observation 64 is not charged as an outlier any more, but a new outlier in the second study group, observation 49, appeared.

**Fig. 2:**
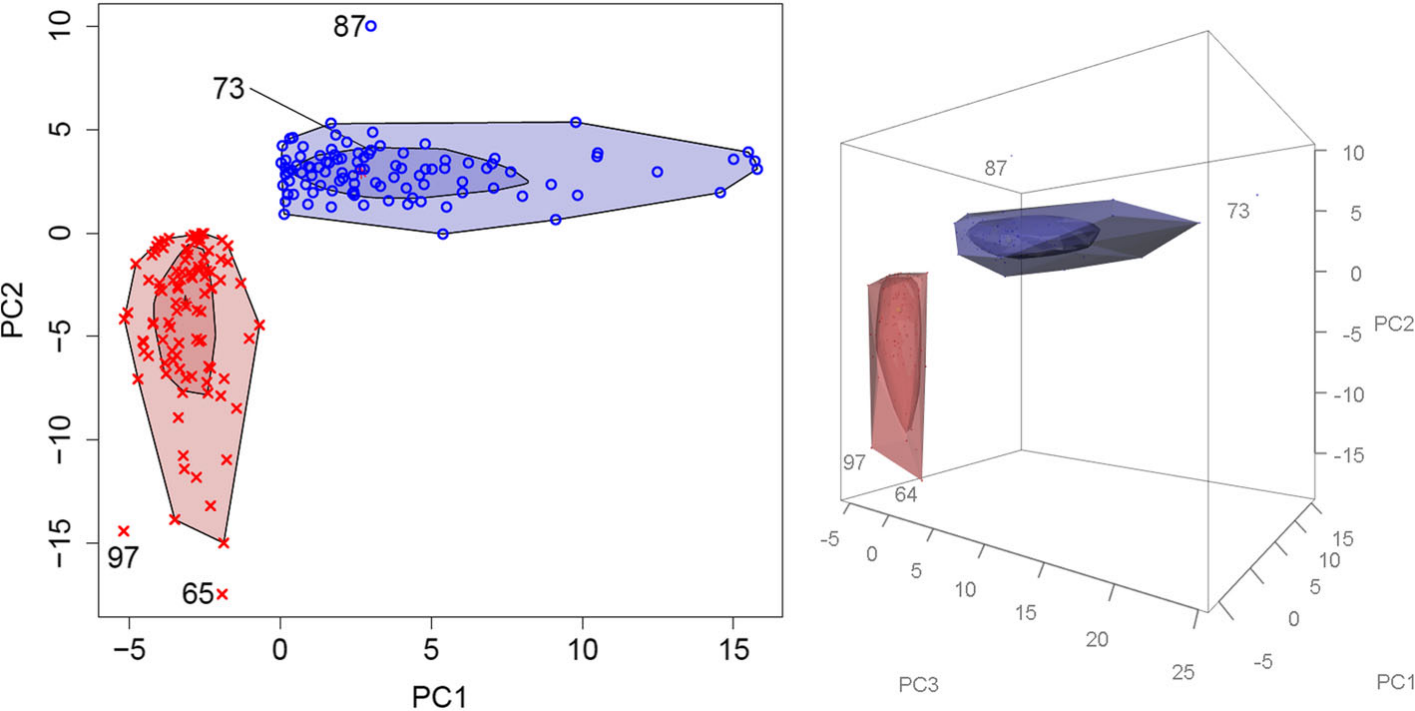
Bagplots and gemplots under non-normaly distributed prinicipal components. In real data, samples are often non-normaly distributed in some principal components. In the blue labelled group samples follow an exponential distribution on the second and third principal components. Thus, sample 73 becomes only an evident outlier in the three-dimensional gemplot

In the second example, where some principal components were not normally distributed, the vanishing and emergence of outliers when switching to more dimensions becomes even much clearer. In the first (blue labeled) study group only observation 87 is detected as outlier in the bagplot approach (Fig. 2). When turning to the gemplot representation, also observation 73 is be detected. In the second study group (red labeled), observations 65 and 97 are detected as outliers in the bagplot approach but they vanish when using gemplots.

### Simulations with artificial gene expression data

Each of the scenarios was simulated in 1000 runs. Setting 1 represented scenarios with only one study group. We first simulated expression data with an overall high correlation between the genes (*τ*=0.01, Additional file 1: Figure S1). Depending on how far away the outliers are from the regular observations (fc=1 to fc=4), the number of detected outliers by the different approaches changes. In general, the boxplot approach (1D) detects more outliers than the bagplot approach (2D) which itself detects more outliers than the gemplot approach (3D). When using the 1D method, boxplots for the first three principal components were inspected. For the 2D method, bagplots for PC1 versus PC2, PC1 versus PC3, and PC2 versus PC3 were used. Then, the union of detected outliers was identified for each method. The 3D method included the first three components anyway. All three approaches only detect outliers that are sufficiently distant from the regular observations, where the 2D and the 3D method require outliers to be more distant than the 1D methods does. I.e., the boxplot approaches appears to be more sensitive than the bagplot which is more sensitive than the gemplot. On the other hand, boxplot and bagplot produce more false detections than the gemplot approach does, i.e. the gemplot outperforms the other tow approaches with respect to specificity. When the overall correlation between the genes is rather low (*τ*=0.05, Additional file 1: Figure S2), the sensitivity of all three approaches increases, while specificity remains nearly the same as with high correlations. The results for scenarios in setting 2, i.e. with two study groups (Additional file 1: Figures S3, S4), are similar to those in setting 1. Again, the highest sensitivity is observed for the 1D approach, while the gemplot approach yields the highest specificity. Likewise, all three methods have a higher sensitivity when the correlation among genes is low and when outliers are clearly distant from the regular observations. Both factors, show no strong effect on the specificity. All simulations show also that the specificity can be improved by the gemplot approach, even if the variance declared by the thirst principal component is less than 10%.

### Analysis of RNA-seq data

**Fig. 3:**
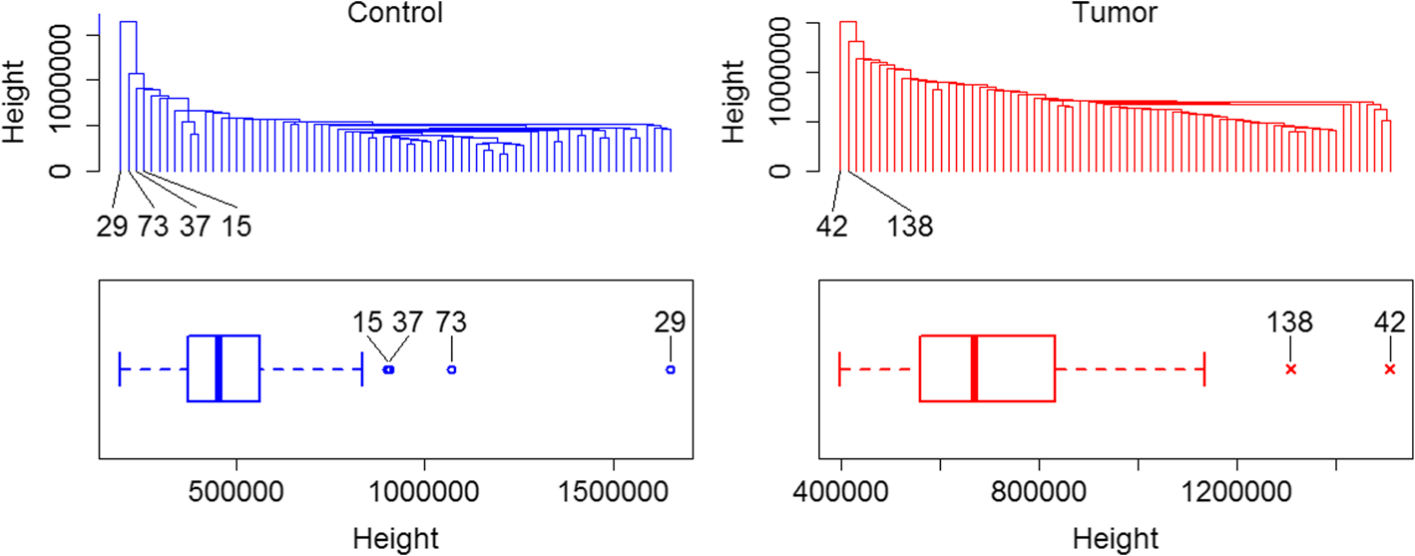
Hierarchical cluster trees and outlier detection RNA-seq data from a kidney tumor study. Clustering was performed using the ‘single linkage’ method which is generally recommended to identify outliers. Usually, outliers are selected by subjective judgment of a researcher as samples on branches that separate locally or by conspicuous height from the remaining branches. To select outliers in a more objective way, boxplots on the height of the branches can be used. A disadvantage of hierarchical cluster trees is that outlier detection and arrangement of the tree depend on whether experimental groups are displayed separately or as a whole

For outlier detection we first employed the approach of hierarchical clustering using the single linkage approach (Fig. 3). Normally, observations on branches that form individual clusters or that separate from the remaining cluster tree by an extreme height are subjectively judged as outliers by visual inspection of the cluster tree. Alternatively to visual inspection, one can transfer the heights of the branches to a boxplot representation to identify outliers in a more objective way. With the latter approach four observations are detected in the control group of the kidney data and two in the tumor group.

**Fig. 4:**
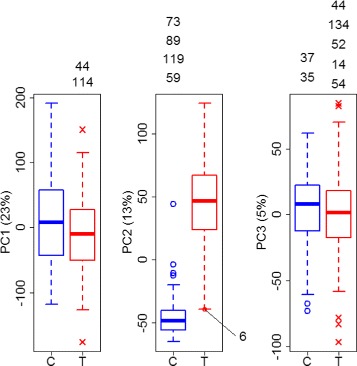
Boxplots of the first three principal components of the kidney data. Group-specific boxplots to detect outliers on the first three principal components. On each dimensional of the principal component space different outliers can be detected

When also using boxplots individually on the first three principal components, large number of outliers is detected (Fig. 4). Of these, observation 37 and 73 in the control group were also detected in the clustering approach, while there was no overlap of findings in the tumor group. Here, it becomes also clear that each principal component can uncover a different set of outliers.

**Fig. 5:**
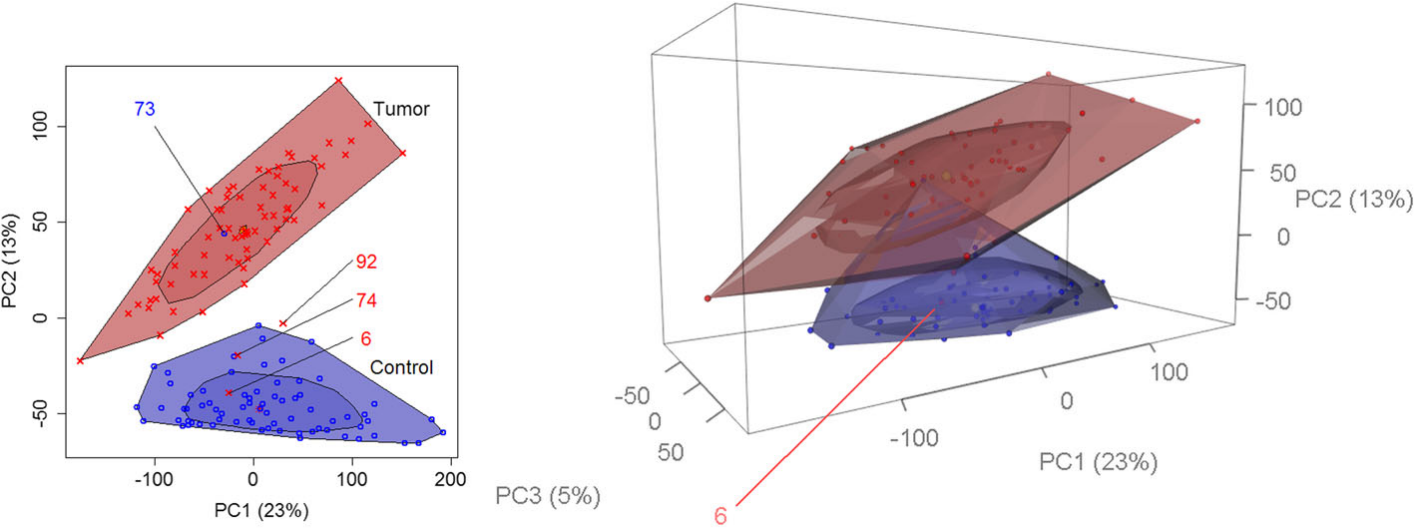
Bagplots and gemplots for representation of the kidney data. In the control group, observation 73 is detected as outlier by the bagplot appraoch but not vanishes when using gemplots. In the tumor group, three outliers are detected using a bagplot of which only observation 6 remains as an outlier in the gemplot approach

When turning to bagplot and gemplot representations of the principal components (Fig. 5), observation 73 is still an outlier in the two-dimensional approach but not in the three-dimensional one. Again, bagplots were used to inspect PC1 versus PC2, PC1 versus PC3, and PC2 versus PC3. Looking at the bagplot of the tumor group the role of correlation between the principal components in this group becomes clear. Observations 74 and 92 are very unlikely to be detected as outliers in boxplots on the first and second principal component. But due to the within-group correlation between the first two components the shape of the bagplot allows for their detection as outliers. Observation 6 appears not as an outlier in the boxplot approach but is detected by the bagplot as well as by the gemplot. Looking at the ‘screeplot’ for the PCA on these data, i.e. the proportion of declared variance per principal component, one can see that there is a sharp bend after PC3 (Additional file 1: Figure S5). According to the ‘elbow method’, one would argue that the principal components behind PC3 don’t provide substantially more information and that they could be omitted for further analysis. Nevertheless, we run the gemplot approach with the first four components to gain 4% more by adding PC4. In that analysis, no observation was flagged as an outlier. Thus, the number of detected outliers reduces in this example when increasing the number of dimensions from 2D to 3D or 4D, i.e. when increasing the number of principal components. The largest number of outliers was detected by boxplots and smallest number by the gemplots. Since in this example the proportion of variance represented was rather small (PC1: 23%, PC2: 13%, PC3: 5%, PC4: 4%), a final decision on outliers might be critical, here.

**Fig. 6:**
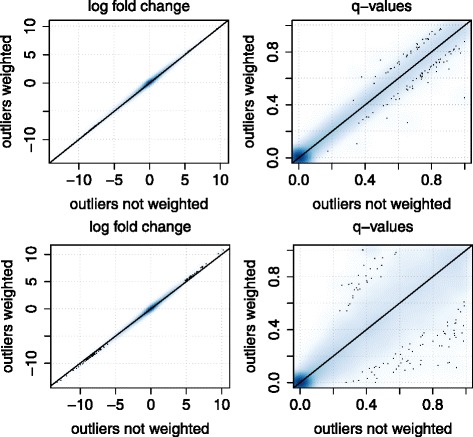
Fold changes and *p*-values with and without outlier weighting. Comparison of fold changes and FDR-adjusted *p*-values from a differential expression analysis between tumor and control samples of the kidney data. Axes show the result with and without weighting of outliers. The upper plots show the effect of only one outlier, the lower plots show the effect of four outliers

In order to demonstrate the impact of outliers we performed a differential expression analysis between tumor and control samples using the R-package ‘limma’ [41]. The functionality of this package allows to downweigh individual observations, and is was shown that this can improve the power of detecting differentially expressed genes [22]. We performed the analysis a) without downweighing outliers, b) by downweighing the four outliers detected by the bagplot approach and c) with downweighing the single outlier, observation 6, identified by the gemplot approach. Figure 6 presents smoothed scatterplots of the log foldchanges resulting from analysis with versus analysis without downweighing outliers as well as scatterplots of FDR-adjusted *p*-values (i.e. *q*-values) of the different approaches. The plots show that only one outlier can have a clear impact on the *p*-values, but only a smaller effect on the fold changes. With four outliers, the effect is even more extreme.

## Discussion

In this work we present a new approach for objective and automated outlier detection in molecular high-throughput data using bagplots and gemplots. The approach is useful for a wide range of data, e.g. gene and protein expression data, methylation data, ChIP data or AP/MS data. All these different data types can first be visualized after dimension reduction by principal component analysis, and bagplots and gemplots can than be applied separately to the observations of each experimental or study group.

Our simulations of gene expression data have shown, that outlier detection with baplots and gemplots on principal components is less sensitive than using boxplots, however, it also reduces significantly the number of false detections. Although two or three components often represent a large proportion of the original variance, it is recommended that researchers explore the variance proportions by scree plots before. A scree plot can help to select an appropriate number of components [35]. In the case that the first three components do not represent a large proportion of the original variance, our approach should be applied with cautiousness.

While we observed in the example of the RNA-seq data that there is a tendency to find less outliers when using more dimensions, we would though recommend to explore the data by at least three principal components with gemplots since more of the original variance is explained than by individual principal components. In particular, our simulations have shown that even if a principal component declares less than 10% of variance, it can contribute to reduce false detections. We observed also in other than the presented data, that gemplots detect less outliers than bagplots or boxplots but this is hard to formalize and depends very much on the shape of the total scatterplot. Thus, an outlier detected by a boxplot of an individual principal component can be fetched back to the set of data points within the fence when adding another principal component. This effect is mainly observed when principal components show a within group correlation in a multiple group setting. One could then argue that principal components don’t represent the correct directions of the largest variances for that group. Analyzing each group individually by PCA would be more appropriate then. On the other hand, one could argue that a simultaneous analysis of all study groups better reflects the variation of gene expression or other high-dimensional measurements in a biological system as a whole, e.g. the variation of different subtypes in a particular cancer disease. A multigroup PCA would for example be more appropriate if outliers come from intermediate subtypes. In any case, researchers should carefully consider whether a single group or multigroup analysis is more appropriate for their data.

Basically, our R-package ‘gemplot’ can calculate outlier detection in more than three dimensions, which becomes, however, very slow for more than four components. In contrast to other approaches that are only based on the individual judgement of the researcher, our approach is more objective and provides an automated detection of outliers. A series of experiments can thus be analysed with the same fix criteria for outlier detection. While some other approaches also provide an automated selection of outliers ([30, 31]), these approaches can only cope with one study group since outlier detection and graphical representation are linked. Thus, our approach is specifically useful when multiple experimental groups are to be analyzed in the same space of principal components. In general, the approach can also be used in other subspaces of the data obtained by dimension reduction, e.g. subspaces derived by multidimensional scaling [42] or factor analysis [43].

As pointed out by Lee et al. [44] and by Ma [45], standard PCA can fail to yield consistent estimators of the loading matrix *A* in high-dimensional settings. Therefore, their approaches for estimating the loadings should also be considered before further exploring the data by bagplots or gemplots.

We also have demonstrated the impact of a small number of outliers on the selection of differentially expressed genes. Similar effects can be assumed for other types of analyses with high-throughput expression data, such as gene set analysis [46–48], classification problems [18–20], and in consequence also for data integration methods of multi-omics data [49, 50]. The concrete handling of outliers detected by our approach depends of course on the specific methods for subsequent analysis. In this regard, another advantage of our approach is that it can be used independently of the methods intended for further analysis.

## Conclusion

We present the gemplot as the three-dimensional version of boxplot and bagplot, respectively. We have demonstrated the usability of the gemplot for outlier detection in molecular, high-dimensional data. In contrast to other methods, our approach allows for simultaneous outlier identification in multiple experimental groups. The presented method is less sensitive than other methods – depending on how extreme the outlying data are – but it produces also less false positives.

## Additional file

Additional file 1 Supplementary Figures Mean numbers of correct detected outliers and incorrect detected outliers in a simulation with artificial gene expression data. **Figure S1.** Scenario with one study group. The four plots show results when the overall correlation between most genes is high (*τ*=0.01). **Figure S2.** Scenario with one study group. The four plots show results when the overall correlation between most genes is low (*τ*=0.05). **Figure S3.** Scenario with two study groups. The four plots show results when the overall correlation between most genes is high (*τ*=0.05) or low (*τ*=0.2) with different fold changes (*f* *c*=1.00 or *f* *c*=1.50). **Figure S4.** Scenario with two study groups. Continuation of Figure S3 for scenarios where outliers are clearly distant from the regular observations (*f* *c*=2.00). **Figure S5.** Screeplot for the principal component analysis of the kidney RNAseq data. (PDF 150 kb)

## Abbreviations

AP/MS: Affinity purification mass spectrometry
ChIP: Chromatin immunoprecipitation
NGS: Next-generation sequencing
PCA: Principal component analysis
RCC: Renal cell carcinomas
