# Supplementary material for: Automated multigroup outlier identification in molecular high-throughput data using bagplots and gemplots
Source: BMC Bioinformatics. 2017 May 2;18:232. doi: 10.1186/s12859-017-1645-5 (PMC5414140; doi:10.1186/s12859-017-1645-5)
Supplement: Additional file 1 — Supplementary Figures Mean numbers of correct detected outliers and incorrect detected outliers in a simulation with artificial gene expression data. Figure S1. Scenario with one study group. The four plots show results when the overall correlation between most genes is high (τ=0.01). Figure S2. Scenario with one study group. The four plots show results when the overall correlation between most genes is low (τ=0.05). Figure S3. Scenario with two study groups. The four plots show results when the overall correlation between most genes is high (τ=0.05) or low (τ=0.2) with different fold changes (f c=1.00 or f c=1.50). Figure S4. Scenario with two study groups. Continuation of Figure S3 for scenarios where outliers are clearly distant from the regular observations (f c=2.00). Figure S5. Screeplot for the principal component analysis of the kidney RNAseq data. (PDF 150 kb) [file 12859_2017_1645_MOESM1_ESM.pdf]

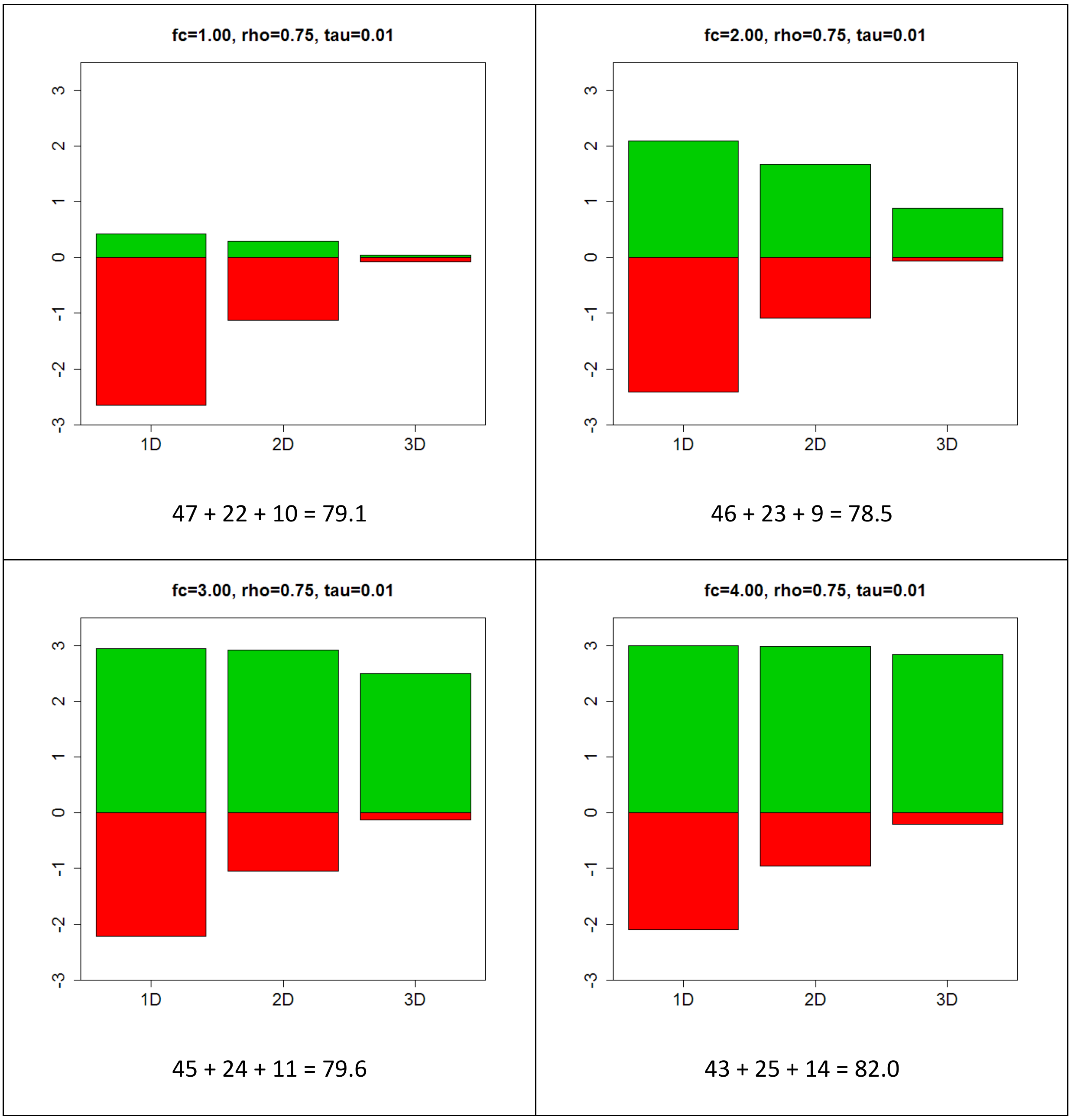

Figure S1: Mean numbers of correct detected outliers (green) and incorrect detected outliers (red) in a simulation with artificial gene expression data. Numbers are given for outlier detection with boxplots (1D), bagplots (2D) and gemplots (3D). Numbers below each plot give the mean percentage of variance declared by each principal component, i.e. %PC1+%PC2+%PC3. The four plots show results when the overall correlation between most genes is high (tau=0.01). Sensitivity and specificity of each approach depends mainly on the distance of outliers to the regular observation, denoted as fold change (fc) here.

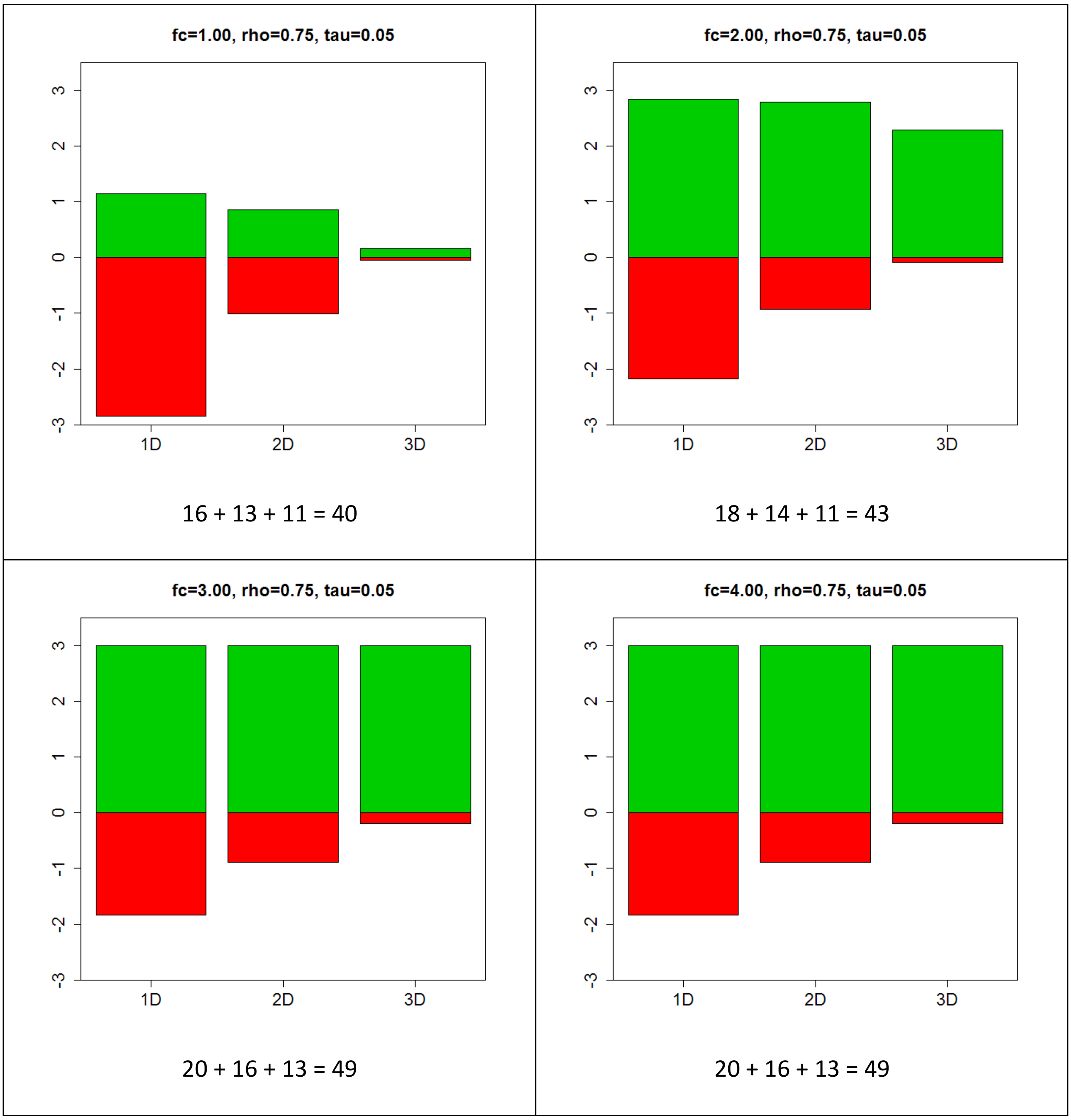

Figure S2: Mean numbers of correct detected outliers (green) and incorrect detected outliers (red) in a simulation with artificial gene expression data. Numbers are given for outlier detection with boxplots (1D), bagplots (2D) and gemplots (3D). Numbers below each plot give the mean percentage of variance declared by each principal component, i.e. %PC1+%PC2+%PC3. The four plots show results when the overall correlation between most genes is low (tau=0.05). Sensitivity and specificity of each approach depends mainly on the distance of outliers to the regular observation, denoted as fold change (fc) here.

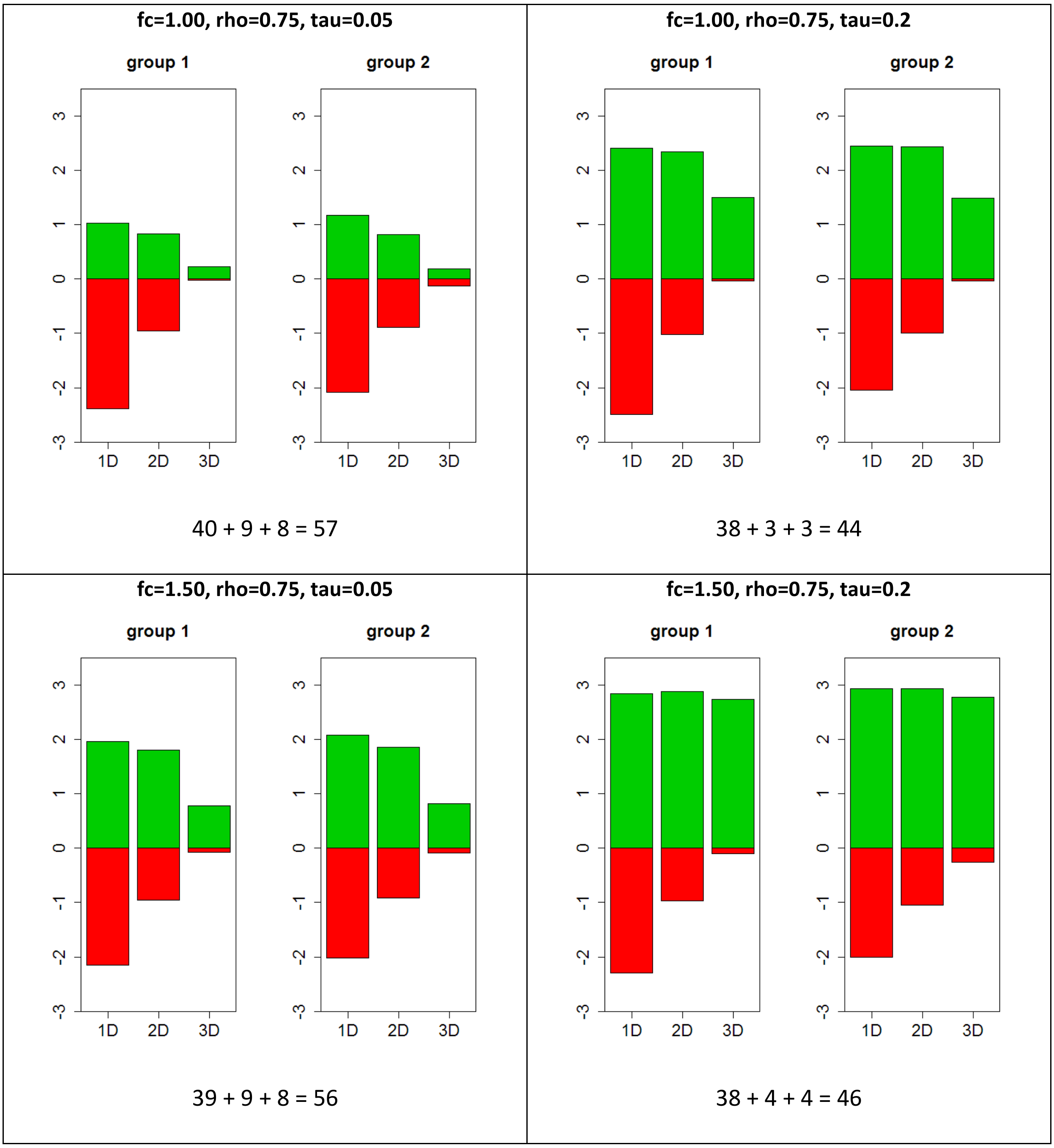

Figure S3: Mean numbers of correct detected outliers (green) and incorrect detected outliers (red) in a simulation with artificial gene expression data of two groups. Numbers are given for outlier detection with boxplots (1D), bagplots (2D) and gemplots (3D). Numbers below each plot give the mean percentage of variance declared by each principal component, i.e. %PC1+%PC2+%PC3. The four plots show results when the overall correlation between most genes is high (tau=0.05) or low (tau=0.2). Sensitivity and specificity of each approach depends mainly on the distance of outliers to the regular observation, denoted as fold change here (fc=1.00 or fc=1.50).

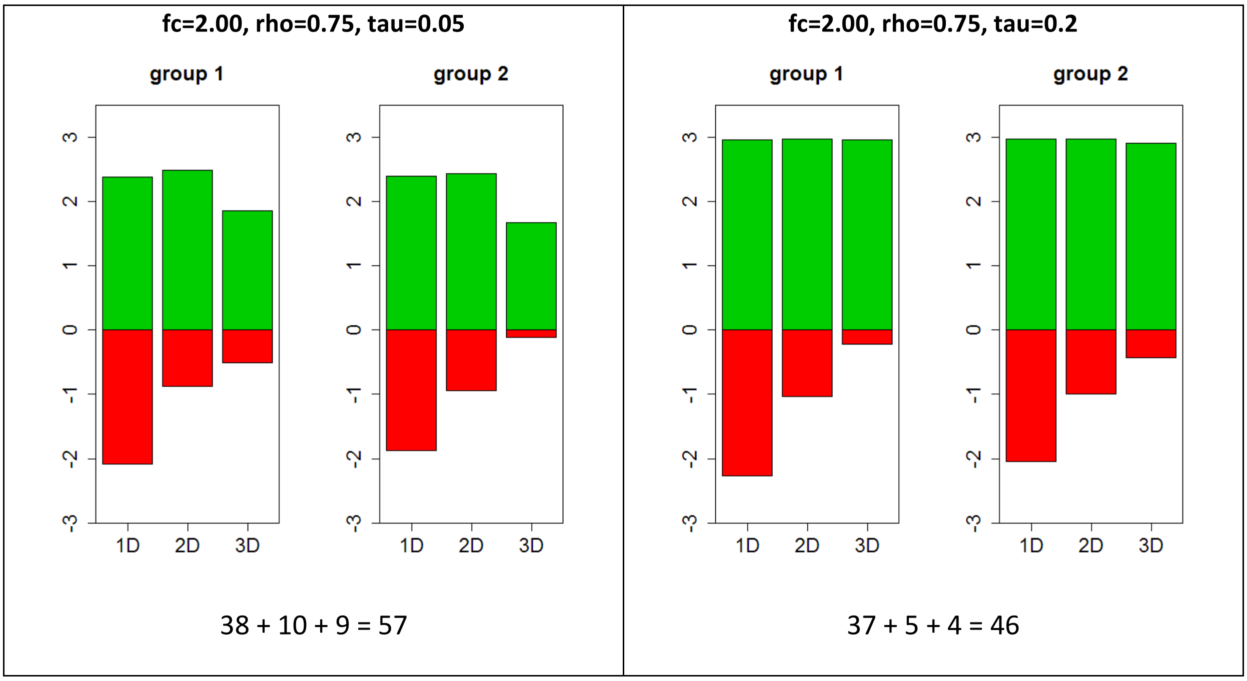

Figure S4: Continuation of Figure S3 for scenarios where outliers are clearly distant from the regular observations (fc=2.00).

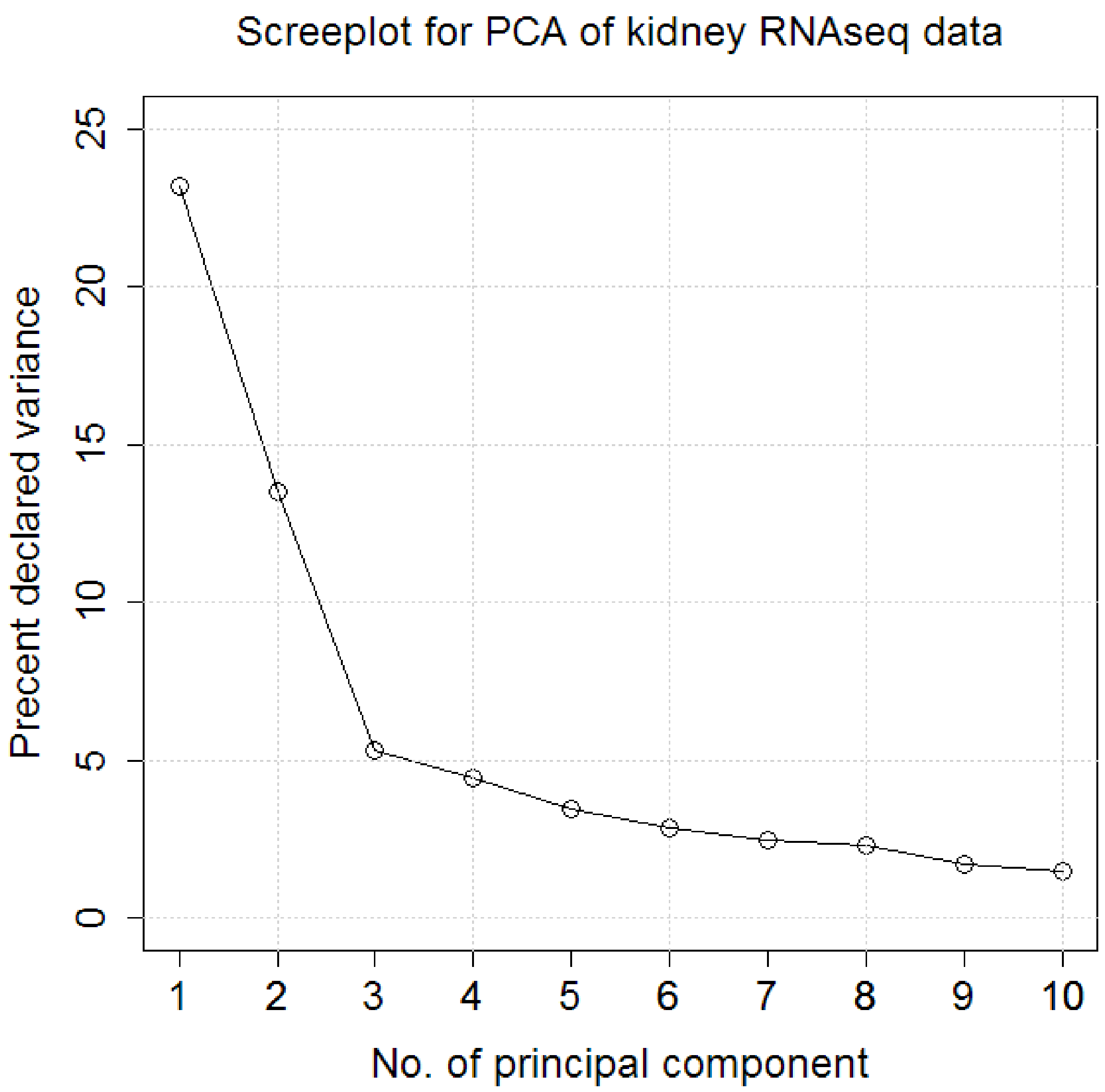

Figure S5: Screeplot for the principal component analysis of the kidney RNAseq data. Principal component higher than PC3 don't contribute to declare much additional variance to the analysis.
